# KB-68A7.1 Inhibits Hepatocellular Carcinoma Development Through Binding to NSD1 and Suppressing Wnt/β-Catenin Signalling

**DOI:** 10.3389/fonc.2021.808291

**Published:** 2022-01-20

**Authors:** Shuhua Zhang, Jianqun Xu, Huan Cao, Mi Jiang, Jun Xiong

**Affiliations:** ^1^ Department of Hepatobiliary Surgery of General Surgery, Union Hospital, Tongji Medical College, Huazhong University of Science and Technology, Wuhan, China; ^2^ Department of Respiratory Medicine, Wuhan Third Hospital, Tongren Hospital of Wuhan University, Wuhan, China

**Keywords:** KB-68A7.1, hepatocellular carcinoma, histone methyltransferase, Wnt/β-catenin signalling, prognosis, progression

## Abstract

Hepatocellular carcinoma (HCC) is one of the most lethal malignancies with extremely poor prognosis. Therefore, revealing the critical molecules involved in HCC progression and prognosis is urgently needed. In this study, through combining public dataset and our cohort, we found a novel prognosis-related long non-coding RNA KB-68A7.1 in HCC. KB-68A7.1 was lowly expressed in HCC, whose low expression was associated with large tumour size, aggressive clinical characteristic, and poor survival. Gain- and loss-of-function assays demonstrated that KB-68A7.1 restricted HCC cellular proliferation, induced HCC cellular apoptosis, and suppressed HCC cellular migration and invasion *in vitro*. Xenograft assays demonstrated that KB-68A7.1 suppressed HCC tumour growth and metastasis *in vivo*. These functional assays suggested KB-68A7.1 as a tumour suppressor in HCC. Histone methyltransferase nuclear receptor binding SET domain-containing protein 1 (NSD1) was found to bind to KB-68A7.1. KB-68A7.1 was mainly distributed in the cytoplasm. The binding of KB-68A7.1 to NSD1 sequestrated NSD1 in the cytoplasm, leading to the reduction in nuclear NSD1 level. Through decreasing nuclear NSD1 level, KB-68A7.1 reduced di-methylation of histone H3 at lysine 36 (H3K36me2) and increased tri-methylation of histone H3 at lysine 27 (H3K27me3) at the promoter of *WNT10B*, a target of NSD1. Thus, KB-68A7.1 repressed *WNT10B* transcription. The expression of WNT10B was negatively correlated with that of KB-68A7.1 in HCC tissues. Through repressing WNT10B, KB-68A7.1 further repressed Wnt/β-catenin signalling. Functional rescue assays showed that overexpression of WNT10B reversed the tumour-suppressive roles of KB-68A7.1, whereas the oncogenic roles of KB-68A7.1 depletion were abolished by Wnt/β-catenin signalling inhibitor. Overall, this study identified KB-68A7.1 as a lowly expressed and prognosis-related lncRNA in HCC, which suppressed HCC progression through binding to NSD1 and repressing Wnt/β-catenin signalling.

## Introduction

Liver cancer is the six most common malignancy and the second leading cause of cancer-related deaths worldwide (1). The relative high mortality indicates that liver cancer is a much malignant cancer, with a 5-year survival of only about 18% (2). Hepatocellular carcinoma (HCC) is the major histological subtype of liver cancer (3). Although surgical resection and some targeted therapies have been applied to fight against HCC, most HCCs remain to have quick progression, which leads to poor prognosis (4–6). Further elucidating the underling molecular mechanisms driving HCC progression would be of clinical importance to develop more efficient therapies against HCC.

Although some genomic mutations have been found in HCC, more genes with aberrant expression accumulate in HCC (7–9). Epigenetic modulation is one of the critical mechanisms contributing to the dysregulated expression of genes (10–12). As one of the epigenetic modulation manners, histone methylation modification exerts multiple effects on gene transcription (13–15). Methylation modification at some specific lysine activates gene transcription, and whereas methylation modification at other lysine represses gene transcription, such as the classical repressive histone marker tri-methylation of histone H3 at lysine 27 (H3K27me3) (16–18). In our previous study, we found an HCC-related histone lysine methyltransferase NSD1 (nuclear receptor binding SET domain-containing protein 1), which catalyses di-methylation of histone H3 at lysine 36 (H3K36me2) (19). Through inducing H3K36me2, NSD1 further decreased H3K27me3 at the promoter of its target genes (19, 20). Our previous study identified WNT10B as a critical downstream target of NSD1 (19). Through upregulating WNT10B expression, NSD1 activates Wnt/β-catenin signalling and exerts oncogenic roles in HCC (19). Except NSD1, other epigenetic modification enzymes are also involved in the progression of HCC (21–23). However, the factors regulating the effects of epigenetic modification enzymes in HCC need further investigation.

Transcriptome and genome studies have demonstrated that only about 2% of human genomes encode proteins, while more than 90% of human genomes transcribe RNAs (24). Thus, most of the RNAs are non-coding RNAs (25, 26). Among these non-coding RNAs, long non-coding RNAs (lncRNAs) are a class of regulatory non-coding RNAs with more than 200 nucleotides in length and no protein-coding potential (27, 28). Aberrant expression of lncRNAs has been found in various diseases (29–32). Moreover, lncRNAs show multiple roles in various pathophysiological processes (33–35). In cancers, many aberrantly expressed lncRNAs were identified as prognostic factors (36). In addition, many lncRNAs exert oncogenic or tumour-suppressive roles (37–41). Although several lncRNAs have been revealed to be involved in HCC (42), the contribution of other lncRNAs and whether they participate in epigenetic modulations in HCC still need further exploration.

To search the lncRNAs contributing to HCC progression, we analyzed The Cancer Genome Atlas (TCGA) liver hepatocellular carcinoma (LIHC) dataset and identified a novel HCC prognosis-related lncRNA KB-68A7.1. KB-68A7.1 has 644 nucleotides in length and is mainly localized in the cytoplasm. The Ensembl ID of KB-68A7.1-encoding gene is ENSG00000274225.1, which is located at chromosome 21q22.3. In this study, we studied the expression pattern, clinical significances, biological roles, and regulatory mechanisms of KB-68A7.1 in HCC. Our finding revealed that KB-68A7.1 is lowly expressed in HCC, and its low expression is correlated with poor survival. KB-68A7.1 has tumour-suppressive roles *via* binding to NSD1, sequestrating NSD1 in the cytoplasm, and repressing Wnt/β-catenin signalling.

## Materials and Methods

### Clinical Samples

Ninety pairs of HCC tissues and adjacent non-cancerous liver tissues were obtained from HCC patients who received surgical excision at Union Hospital, Tongji Medical College, Huazhong University of Science and Technology (Wuhan, China). All samples were frozen in liquid nitrogen and stored in −80°C freezer until use. The study was performed following the ethical principles for medical research involving human subjects of the Helsinki Declaration and approved by the Ethic Committee of Union Hospital, Tongji Medical College, Huazhong University of Science and Technology (Wuhan, China). Written informed consents were acquired from all patients.

### Cell Culture and Grouping

Human immortalized liver cell line (THLE-3) and human HCC cell lines (SNU-398 and SK-HEP-1) were obtained from the American Type Culture Collection (ATCC). Human HCC cell line (HuH-7) was obtained from Chinese Academy of Science Cell Bank (Shanghai, China). SNU-398 and HuH-7 cells were both epithelial, and SK-HEP-1 cell was endothelial. THLE-3 was cultured using BEGM Bullet Kit (Lonza, Walkersville, MD, USA) in accordance with the provided protocol. SNU-398, SK-HEP-1, and HuH-7 were cultured in Roswell Park Memorial Institute (RPMI) 1640 medium (Invitrogen, Carlsbad, CA USA), Eagle’s Minimum Essential Medium (Invitrogen), and Dulbecco’s modified Eagle’s medium (Invitrogen), respectively, all of which were supplemented with 10% foetal bovine serum (FBS, Invitrogen). Cells were maintained at 37°C in an incubator with 5% CO_2_.

To obtain HCC cells with KB-68A7.1 stable overexpression, KB-68A7.1 overexpression lentivirus was purchased from GenePharma (Shanghai, China), which infected SNU-398 and HuH-7 cells. Ninety-six hours later, infected cells were treated with 2 μg/ml of puromycin to select SNU-398 and HuH-7 cells with KB-68A7.1 stable overexpression. To obtain HCC cells with KB-68A7.1 and WNT10B concurrently stable overexpression, WNT10B overexpression plasmid (iGene Biotechnology, Guangzhou, China) was transfected into SNU-398 cells with KB-68A7.1 stable overexpression. Forty-eight hours later, transfected cells were treated with 2 μg/ml of puromycin and 1,000 μg/ml of neomycin to select SNU-398 cells with KB-68A7.1 and WNT10B concurrently stable overexpression. To obtain HCC cells with KB-68A7.1 stable silencing, KB-68A7.1-specific short hairpin RNA (shRNA) lentiviruses were purchased from GenePharma (Shanghai, China), which infected SK-HEP-1 and SNU-398 cells. Ninety-six hours later, infected cells were treated with 2 μg/ml of puromycin to select SK-HEP-1 and SNU-398 cells with KB-68A7.1 stable depletion. Scrambled non-targeting shRNA lentivirus was used as negative control. The shRNA sequences were as follows: 5′-GATCCGGAACTCCAATGGGAAACTAATTCAAGAGATTAGTTTCCCATTGGAGTTCCTTTTTTG-3′ (sense) and 5′-AATTCAAAAAAGGAACTCCAATGGGAAACTAATCTCTTGAATTAGTTTCCCATTGGAGTTCCG-3′ (antisense) for shRNA-KB-68A7.1-1; 5′-GATCCGATTCATACGGCACATCTTGTTTCAAGAGAACAAGATGTGCCGTATGAATCTTTTTTG-3′ (sense) and 5′-AATTCAAAAAAGATTCATACGGCACATCTTGTTCTCTTGAAACAAGATGTGCCGTATGAATCG-3′ (antisense) for shRNA-KB-68A7.1-2; 5′-GATCCGTTCTCCGAACGTGTCACGTTTCAAGAGAACGTGACACGTTCGGAGAACTTTTTTG-3′ (sense) and 5′-AATTCAAAAAAGTTCTCCGAACGTGTCACGTTCTCTTGAAACGTGACACGTTCGGAGAACG-3′ (antisense) for shRNA control.

### RNA Isolation, Reverse Transcription, and Quantitative Polymerase Chain Reaction

Total RNA was extracted from indicated tissues and cells using Trizol reagent (Invitrogen). The quality and concentration of RNA were assessed using the NanoDrop 2000 Spectrophotometer (Thermo Scientific, Waltham, MA, USA). RNA was subjected to reverse transcription (RT) using the PrimeScript II First-Strand cDNA Synthesis Kit (Takara, Dalian, China). The cDNA was further subjected to quantitative polymerase chain reaction (qPCR) using the TB Green Premix Ex Taq II (Takara) on StepOnePlus Real-Time PCR System (Thermo Scientific). Glyceraldehyde 3-phosphate dehydrogenase (GAPDH) was employed as internal control. The primer sequences were as follows: 5′-ACGAGTGAACAAAAGACGC-3′ (sense) and 5′-CAAACCAGAGGGGAAAATG-3′ (antisense) for KB-68A7.1; 5′-GAAGGTGAAGGTCGGAGTC-3′ (sense) and 5′-GAAGATGGTGATGGGATTTC-3′ (antisense) for GAPDH; 5′-GGAGCAGAAAACAGCAGG-3′ (sense) and 5′-GGAAGGGGTCAGGAGAAA-3′ (antisense) for MALAT1; 5′-GGAATGGGGTGGCTGTAAC-3′ (sense) and 5′-CGGTTGTGGGTATCAATGAAG-3′ (antisense) for WNT10B; 5′-CTTCCCCTACCCTCTCAA-3′ (sense) and 5′-CGATTTCTTCCTCATCTTCT-3′ (antisense) for Myc; 5′-ACAACTTCCTGTCCTACTACC-3′ (sense) and 5′-TCCTCTTCCTCCTCCTCG-3′ (antisense) for cyclin D1.

### Cell Proliferation Assays

Cellular proliferation ability was assessed by the Cell Counting Kit-8 (CCK-8) and 5-ethynyl-2′-deoxyuridine (EdU) incorporation assays. CCK-8 assay was performed as we previously described (19). Briefly, 5,000 indicated HCC cells were seeded into 96-well plate. After culture for 72 h, cellular proliferation ability was measured using CCK-8 reagent (Dojindo Laboratories, Kumamoto, Japan), which was indicated by the optical density value at 450 nm. EdU incorporation assay was performed using the Cell-Light EdU Apollo 567 *In Vitro* Kit (RiboBio, Guangzhou, China) as previously described (43).

### Cell Apoptosis Assays

Cellular apoptosis was assessed by terminal deoxynucleotidyl transferase (TdT)-mediated dUTP nick end labelling (TUNEL) and caspase-3 activity assays. TUNEL assay was performed using the One Step TUNEL Apoptosis Assay Kit (Beyotime Biotechnology, Shanghai, China). Caspase-3 activity assay was performed using the Caspase-3 Activity Assay Kit (Cell Signalling Technology, Danvers, MA, USA) following the provided protocol.

### Cell Migration and Invasion Assays

Cellular migration and invasion abilities were assessed by Transwell migration assay and Transwell invasion assay, respectively, as we previously described (19). Briefly, indicated HCC cells re-suspended in FBS-free medium were added to the upper chamber of Transwell inserts (pore size, 8 μm; Corning, NY, USA) coated with or without Matrigel. Medium containing 20% FBS were added to the lower chamber. After culture for 24 h, the cells in the upper chamber were removed. The cells on the outer layer of the membranes were fixed, stained, photographed, and counted.

### Xenografts Experiments

Xenograft experiments were performed with the approval of the Institutional Animal Care and Use Committee of Union Hospital, Tongji Medical College, Huazhong University of Science and Technology (Wuhan, China). Five-week-old male athymic BALB/c nude mice were used. A total of 2 × 10^6^ indicated cells were subcutaneously inoculated into the flanks of mice. Subcutaneous xenograft volumes were measured every 7 days using a calliper and calculated by the formula V = 0.5 × a × b^2^ (a, the long diameter; b, the short diameter). On the 21st day after inoculation, subcutaneous xenografts were resected and weighed. Subcutaneous xenografts were subjected to immunohistochemistry (IHC) staining using the primary antibodies against Ki67 (ab15580, 1:200, Abcam, Cambridge, MA, USA), PCNA (ab92552, 1:200, Abcam), or cleaved caspase-3 (#9661, 1;200, Cell Signalling Technology). Furthermore, subcutaneous xenografts were also subjected to TUNEL assays using the One Step TUNEL Apoptosis Assay Kit (Beyotime Biotechnology) following the provided instruction. A total of 2 × 10^6^ indicated cells were intrasplenically injected into the mice to construct liver metastasis model. On the 35th day after injection, the livers were resected and subjected to haematoxylin–eosin (H&E) staining.

### RNA Fluorescence *In Situ* Hybridization

For *in situ* detection of KB-68A7.1, the probes against KB-68A7.1 were designed and synthesized by Advanced Cell Diagnostics (Newark, CA, USA). The hybridization and fluorescence detection were conducted using the RNAscope Fluorescent Multiplex Detection Kit (Advanced Cell Diagnostics) following the provided manual. The subcellular localization of KB-68A7.1 was detected using the Leica Confocal Microscope TCS SP8 (Leica, Wetzlar, Germany).

### Purification of Cytoplasmic and Nuclear RNA

Cytoplasmic and nuclear RNA was purified using the PARIS Kit (Thermo Scientific) in accordance with the provided manual. The purified RNA was measured using reverse transcription qPCR (RT-qPCR) as above described.

### RNA Immunoprecipitation Assay

RNA immunoprecipitation (RIP) assay was conducted using the Magna RIP RNA-Binding Protein Immunoprecipitation Kit (Millipore, Burlington, MA, USA) and NSD1-specific antibody (H00064324-M08, 5 µg per reaction, Novus Biologicals, Centennial, CO, USA). Enrichment of RNA was detected using RT-qPCR as above described.

### RNA–Protein Pull-Down Assay

KB-68A7.1 full-length sequences were PCR amplified with the primers 5′-GCTCTAGATAGACCACAGGGAGGCCTG-3′ (sense) and 5′-GGGGTACCACTTGTCAATCGAGATTTTAA-3′ (antisense). The products were further subcloned into the Xba*I* and Kpn*I* sites of pSPT19 vector (Roche, Basel, Switzerland) to construct KB-68A7.1 *in vitro* transcription vector. KB-68A7.1 was *in vitro* transcribed from this constructed vector using T7 RNA polymerase (Roche). After purification, KB-68A7.1 RNA was end-labelled with desthiobiotin using the Pierce RNA 3′ End Desthiobiotinylation Kit (Thermo Scientific). Pull-down reactions were further conducted using the desthiobiotinylated RNA and the Pierce Magnetic RNA-Protein Pull-Down Kit (Thermo Scientific) following the provided manual. The enriched proteins were detected by Western blot.

### Western Blot

Total protein was extracted from indicated cells using radioimmunoprecipitation assay (RIPA) lysis buffer (Beyotime). Nuclear protein was extracted from indicated cells using the Nuclear and Cytoplasmic Protein Extraction Kit (Beyotime). Protein concentration was quantified using the Enhanced BCA Protein Assay Kit (Beyotime). Next, the proteins were separated by 7.5% or 10% sodium dodecyl sulphate-polyacrylamide gel electrophoresis, followed by transferring onto a polyvinylidene fluoride membrane (Millipore). After being sealed, the membranes were incubated with primary antibodies against NSD1 (H00064324-M08, 1:500, Novus Biologicals), histone H3 (AF0863, 1:1,000, Affinity, Changzhou, Jiangsu, China), WNT10B (ab70816, 1:1,000, Abcam), GAPDH (60004-1-Ig, 1:10,000, Proteintech, Chicago, IN, USA), or β-catenin (#8480, 1:1,000, Cell Signalling Technology) overnight at 4°C. After washes, the membranes were further incubated with IRDye 680RD Goat Anti-Mouse IgG Secondary Antibody (926-68070, 1:10,000, Li-Cor Biosciences, Lincoln, NE, USA) or IRDye 800CW Goat Anti-Rabbit IgG Secondary Antibody (926-32211, 1:10,000, Li-Cor). Lastly, the membranes were scanned using the Odyssey infrared scanner (Li-Cor). Histone H3 and GAPDH were employed as loading controls for nuclear protein and total protein, respectively.

### Immunofluorescence

Subcellular distribution of NSD1 was assessed by immunofluorescence (IF). Indicated cells were cultured and fixed on 12 × 12 mm glass slides. After being incubated with primary antibody against NSD1 (ab222145, 4 μg/ml, Abcam) overnight at 4°C, the slides were washed and further incubated with goat anti-rabbit IgG cross-adsorbed secondary antibody, Alexa Fluor 594 (A-11012, 1:1,000, Invitrogen). Then, the cell nuclei were stained with 4′,6-diamidino-2-phenylindole (DAPI). Lastly, the slides were photographed with the Leica Confocal Microscope TCS SP8 (Leica).

### Chromatin Immunoprecipitation

Chromatin immunoprecipitation (ChIP) assays were conducted using the ChIP Kit (ab500, Abcam) and primary antibodies against H3K36me2 (ab176921, 2 µg, Abcam) or H3K27me3 (ab192985, 2 µg, Abcam) following the provided manual. The enriched DNA was detected using qPCR as above described with the primers 5′-GTCTGGCTCCATCCTCATCT-3′ (sense) and 5′-CTCTCTCACACACCCTCTCC-3′ (antisense) for *WNT10B* promoter.

### Dual Luciferase Reporter Assay

Wnt/β-catenin reporter TOPflash (Addgene, Watertown, MA, USA), which expresses Firefly luciferase, was co-transfected with pRL-TK (Promega, Madison, WI, USA), which expresses Renilla luciferase into indicated cells using Lipofectamine 3000 (Invitrogen). Forty-eight hours later, the luciferase activities were detected by the Dual-Luciferase Reporter Assay System (Promega) in accordance with the provided manual. Renilla luciferase activity was employed as internal control.

### Statistical Analysis

Statistical analyses were conducted using GraphPad Prism v6.0 (San Diego, CA, USA). As shown in figures and table legends, Wilcoxon matched-pairs signed-rank test, log-rank test, one-way ANOVA followed by Dunnett’s multiple comparisons test, two-tailed unpaired t-test, Spearman correlation analysis, and Pearson chi-square test were performed. p < 0.05 was set as the threshold for statistical significance.

## Results

### Low Expression of KB-68A7.1 Was Associated With Poor Survival in HCC

**Figure 1:**
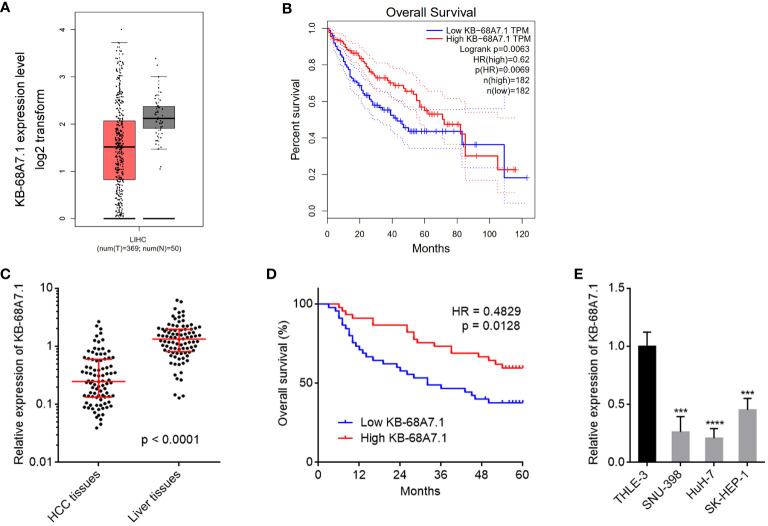
The expression and prognostic relevance of KB-68A7.1 in HCC. **(A)** KB-68A7.1 expression in HCC tissues and normal liver tissues from TCGA LIHC dataset was analysed by GEPIA. **(B)** The correlation between KB-68A7.1 expression in HCC tissues and overall survival according to TCGA LIHC dataset was analysed by GEPIA. **(C)** KB-68A7.1 expression in 90 pairs HCC tissues and adjacent liver tissues was measured by RT-qPCR. p < 0.0001 by Wilcoxon matched-pairs signed-rank test. **(D)** Kaplan–Meier survival curves of 90 HCC patients stratified by KB-68A7.1 expression. Median KB-68A7.1 expression was used as cutoff. HR = 0.4829, p = 0.0128 by log-rank test. **(E)** KB-68A7.1 expression in immortalized liver cell line THLE-3 and HCC cell lines SNU-398, HuH-7, and SK-HEP-1 was measured by RT-qPCR. Results are shown as mean ± SD based on three independent experiments. ***p < 0.001, ****p < 0.0001 by one-way ANOVA followed by Dunnett’s multiple comparisons test.

The expression and clinical relevance of KB-68A7.1 in HCC were assessed *via* investigating TCGA liver hepatocellular carcinoma (LIHC) dataset using the GEPIA (http://gepia.cancer-pku.cn/index.html) (44). The results revealed that KB-68A7.1 was lowlier expressed in HCC tissues than that in normal liver tissues (**Figure 1A**). The TCGA LIHC dataset also presented that low expression of KB-68A7.1 was associated with short overall survival (**Figure 1B**). Furthermore, the expression and clinical relevance of KB-68A7.1 were assessed in HCC tissues obtained in our hospital. The results once again revealed that KB-68A7.1 was lowlier expressed in HCC tissues than that in matched adjacent liver tissues (**Figure 1C**). Encouragingly, Kaplan–Meier survival analysis once again revealed that low expression of KB-68A7.1 was associated with short overall survival in our HCC cohort (**Figure 1D**). Correlation analyses between KB-68A7.1 expression levels and clinicopathological characteristics revealed that low expression of KB-68A7.1 was associated with large tumour size, encapsulation incomplete, vascular invasion, and advanced clinical stage (**Table 1**). The expression of KB-68A7.1 in immortalized liver cell line THLE-3 and HCC cell lines SNU-398, HuH-7, and SK-HEP-1 were assessed using RT-qPCR. We found that the expression of KB-68A7.1 was lower in HCC cell lines than that in immortalized liver cell line (**Figure 1E**). Taken together, these results demonstrated that KB-68A7.1 was lowly expressed in HCC, and its low expression was associated with large tumour size, aggressive clinical characteristics, and poor survival.

**Table 1:** Correlation between KB-68A7.1 expression levels and clinicopathological characteristics of 90 HCC patients.

Feature	KB-68A7.1 expression		χ^2^	p-value
	Low	High		
Age			0.741	0.389
>50	29	25		
≤50	16	20		
Gender			0.653	0.419
Male	35	38		
Female	10	7		
HBs antigen			0.559	0.455
Positive	33	36		
Negative	12	9		
Liver cirrhosis			1.113	0.291
With	19	24		
Without	26	21		
AFP (µg/L)			0.963	0.327
>20	36	32		
≤20	9	13		
Grade			1.538	0.215
G1–G2	4	8		
G3–G4	41	37		
Tumour size (cm)			5.657	0.017
>5	33	22		
≤5	12	23		
Encapsulation			4.731	0.030
Complete	7	16		
Not complete/no	38	29		
Vascular invasion			6.403	0.011
Absent	16	28		
Present	29	17		
TNM staging			10.041	0.018
I	3	11		
II	10	15		
III	30	19		
IV	2	0		

p-value was acquired by Pearson’ chi-square tests.

### KB-68A7.1 Restricted HCC Cellular Proliferation, Migration, and Invasion, and Induced HCC Cellular Apoptosis

**Figure 2:**
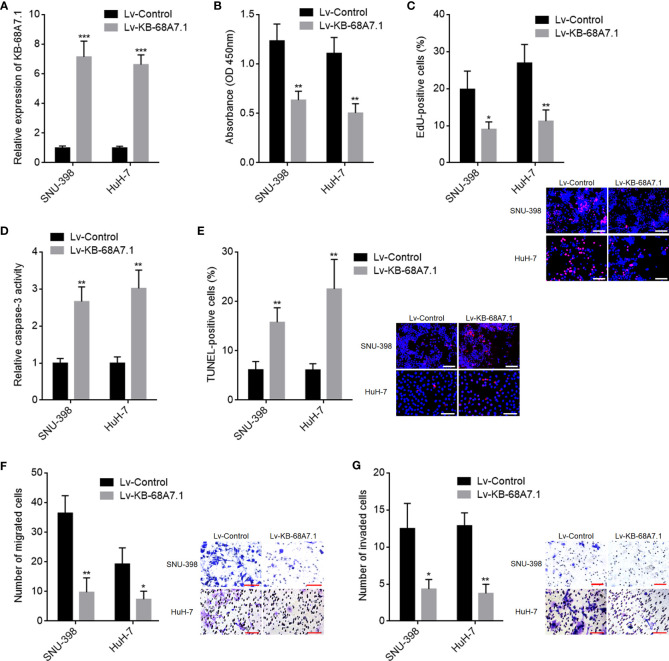
The roles of KB-68A7.1 overexpression in HCC cellular proliferation, apoptosis, migration, and invasion. **(A)** KB-68A7.1 expression in SNU-398 and HuH-7 cells with KB-68A7.1 stable overexpression or control was measured by RT-qPCR. **(B)** Cellular proliferation of SNU-398 and HuH-7 cells with KB-68A7.1 overexpression or control was measured by CCK-8 assay. **(C)** Cellular proliferation of SNU-398 and HuH-7 cells with KB-68A7.1 overexpression or control was measured by EdU incorporation assay. Representative images are shown on low right. Red colour indicates EdU-positive and proliferative cells. Scale bars = 100 µm. **(D)** Cellular apoptosis of SNU-398 and HuH-7 cells with KB-68A7.1 overexpression or control was measured by caspase-3 activity assay. **(E)** Cellular apoptosis of SNU-398 and HuH-7 cells with KB-68A7.1 overexpression or control was measured by TUNEL assay. Representative images are shown on the right. Red colour indicates TUNEL-positive and apoptotic cells. Scale bars = 100 µm. **(F)** Cellular migration of SNU-398 and HuH-7 cells with KB-68A7.1 overexpression or control was measured by Transwell migration assay. Representative images are shown on the right. Scale bars = 100 µm. **(G)** Cell invasion of SNU-398 and HuH-7 cells with KB-68A7.1 overexpression or control was measured by Transwell invasion assay. Representative images are shown on the right. Scale bars = 100 µm. Results are shown as mean ± SD based on three independent experiments. *p < 0.05, **p < 0.01, ***p < 0.001 by two-tailed unpaired t-test.

Next, the roles of KB-68A7.1 in HCC were explored. KB-68A7.1 was stably overexpressed in SNU-398 and HuH-7 cells *via* infecting KB-68A7.1 overexpression lentivirus. The expression efficiency was validated by RT-qPCR (**Figure 2A**). CCK-8 and EdU incorporation assays were undertaken to assess cellular proliferation, which demonstrated that ectopic expression of KB-68A7.1 inhibited cellular proliferation ability in both SNU-398 and HuH-7 cells (**Figures 2B, C**). Caspase-3 activity assay and TUNEL assay were undertaken to assess cellular apoptosis, which demonstrated that ectopic expression of KB-68A7.1 induced cellular apoptosis in both SNU-398 and HuH-7 cells (**Figures 2D, E**). Transwell migration and invasion assays were undertaken to assess cellular migration and invasion respectively, which demonstrated that ectopic expression of KB-68A7.1 inhibited cellular migration and invasion abilities in both SNU-398 and HuH-7 cells (**Figures 2F, G**). Collectively, these results demonstrated that KB-68A7.1 restricted HCC cellular proliferation, induced HCC cellular apoptosis, and restricted HCC cellular migration and invasion.

### KB-68A7.1 Depletion Promoted HCC Cellular Proliferation, Migration, and Invasion, and Repressed HCC Cellular Apoptosis

**Figure 3:**
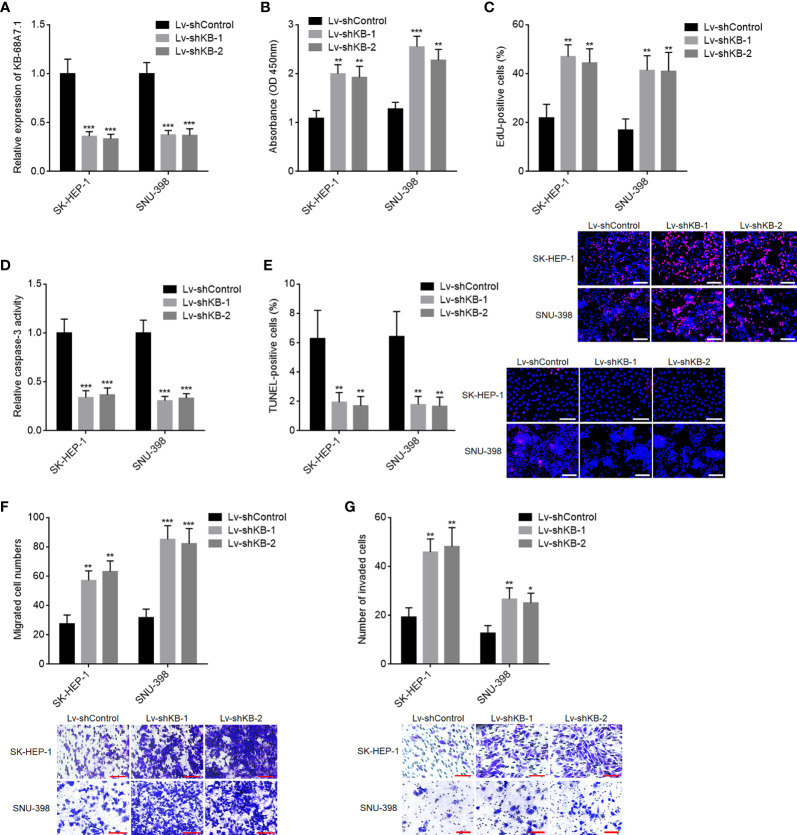
The roles of KB-68A7.1 silencing in HCC cellular proliferation, apoptosis, migration, and invasion. **(A)** KB-68A7.1 expression in SK-HEP-1 and SNU-398 cells with KB-68A7.1 stable silencing or control was measured by RT-qPCR. **(B)** Cellular proliferation of SK-HEP-1 and SNU-398 cells with KB-68A7.1 silencing or control was measured by CCK-8 assay. **(C)** Cellular proliferation of SK-HEP-1 and SNU-398 cells with KB-68A7.1 silencing or control was measured by EdU incorporation assay. Representative images are shown on low right. Red colour indicates EdU-positive and proliferative cells. Scale bars = 100 µm. **(D)** Cellular apoptosis of SK-HEP-1 and SNU-398 cells with KB-68A7.1 silencing or control was measured by caspase-3 activity assay. **(E)** Cellular apoptosis of SK-HEP-1 and SNU-398 cells with KB-68A7.1 silencing or control was measured by TUNEL assay. Representative images are shown on the right. Red colour indicates TUNEL-positive and apoptotic cells. Scale bars = 100 µm. **(F)** Cellular migration of SK-HEP-1 and SNU-398 cells with KB-68A7.1 silencing or control was measured by Transwell migration assay. Representative images are shown below. Scale bars = 100 µm. **(G)** Cell invasion of SK-HEP-1 and SNU-398 cells with KB-68A7.1 silencing or control was measured by Transwell invasion assay. Representative images are shown below. Scale bars = 100 µm. Results are shown as mean ± SD based on three independent experiments. *p < 0.05, **p < 0.01, ***p < 0.001 by one-way ANOVA followed by Dunnett’s multiple comparisons test.

To further explore the roles of KB-68A7.1 in HCC, KB-68A7.1 was stably silenced in SK-HEP-1 and SNU-398 cells *via* infecting KB-68A7.1 shRNA lentivirus (**Figure 3A**). CCK-8 and EdU incorporation assays demonstrated that silencing of KB-68A7.1 enhanced cellular proliferation ability in both SK-HEP-1 and SNU-398 cells (**Figures 3B, C**). Caspase-3 activity assay and TUNEL assay demonstrated that silencing of KB-68A7.1 repressed cellular apoptosis in both SK-HEP-1 and SNU-398 cells (**Figures 3D, E**). Transwell migration and invasion assays demonstrated that silencing of KB-68A7.1 enhanced cellular migration and invasion abilities in both SK-HEP-1 and SNU-398 cells (**Figures 3F, G**). Furthermore, KB-68A7.1 was also silenced in immortalized liver cell line THLE-3 (**Supplementary Figure S1A**). CCK-8 and EdU incorporation assays demonstrated that silencing of KB-68A7.1 also enhanced cellular proliferation ability in THLE-3 cells (**Supplementary Figures S1B, C**). Caspase-3 activity assay and TUNEL assay demonstrated that silencing of KB-68A7.1 also repressed cellular apoptosis in THLE-3 cells (**Supplementary Figures S1D, E**). Transwell migration and invasion assays demonstrated that silencing of KB-68A7.1 also enhanced cellular migration and invasion abilities in THLE-3 cells (**Supplementary Figures S1F, G**). Taken together, these data further supported the tumour-suppressive roles of KB-68A7.1 in HCC.

### KB-68A7.1 Suppressed HCC Tumour Growth and Metastasis

**Figure 4:**
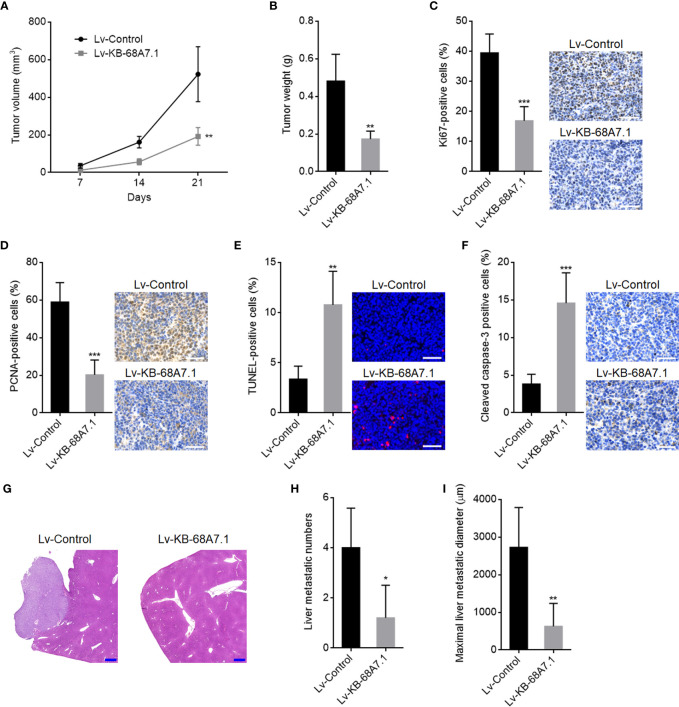
The roles of KB-68A7.1 in HCC tumour growth and metastasis. **(A, B)** SNU-398 cells with KB-68A7.1 stable overexpression or control were subcutaneously injected into nude mice. Subcutaneous xenograft volumes were measured every 7 days **(A)**. Subcutaneous xenografts were resected and weighed at the 21st day after inoculation **(B)**. **(C)** Subcutaneous xenografts were subjected to Ki67 IHC staining. Scale bars = 50 µm. **(D)** Subcutaneous xenografts were subjected to PCNA IHC staining. Scale bars = 50 µm. **(E)** Subcutaneous xenografts were subjected to TUNEL assays. Scale bars = 50 µm. **(F)** Subcutaneous xenografts were subjected to cleaved caspase-3 IHC staining. Scale bars = 50 µm. **(G**–**I)** SNU-398 cells with KB-68A7.1 stable overexpression or control were intrasplenically injected into nude mice to construct liver metastasis model. At the 35th day after injection, the livers were resected and subjected to H&E staining. Scale bars = 500 µm **(G)**. The number **(H)** and size **(I)** of liver metastatic nodules were counted. Results are shown as mean ± SD based on n = 5 mice in each group. *p < 0.05, **p < 0.01, ***p < 0.001 by two-tailed unpaired t-test.

The roles of KB-68A7.1 in HCC were further explored using xenograft models *in vivo*. SNU-398 cells with KB-68A7.1 stable overexpression, or control were subcutaneously injected into nude mice. The subcutaneous tumour growth curve showed that the xenografts formed by SNU-398 cells with KB-68A7.1 overexpression grew much slower than those formed by control SNU-398 cells (**Figure 4A**). On the 21st day after injection, the subcutaneous xenografts were resected and weighed, which once again showed that ectopic expression of KB-68A7.1 suppressed tumour growth *in vivo* (**Figure 4B**). The resected xenografts were subjected to proliferation marker Ki67 and PCNA IHC staining, which presented that KB-68A7.1 repressed cellular proliferation *in vivo* (**Figures 4C, D**). Cellular apoptosis was assessed by TUNEL assay and cleaved caspase-3 IHC staining, which presented that KB-68A7.1 promoted cellular apoptosis *in vivo* (**Figures 4E, F**). To explore the roles of KB-68A7.1 in HCC metastasis *in vivo*, SNU-398 cells with KB-68A7.1 stable overexpression or control were intrasplenically injected into nude mice to construct the liver metastasis model, which presented that SNU-398 cells with KB-68A7.1 overexpression formed much fewer and smaller liver metastatic nodules than control SNU-398 cells (**Figures 4G–I**). Collectively, these results demonstrated that KB-68A7.1 restricted HCC tumour growth and metastasis *in vivo*.

### KB-68A7.1 Bound to NSD1 and Reduced the Nuclear Distribution of NSD1

**Figure 5:**
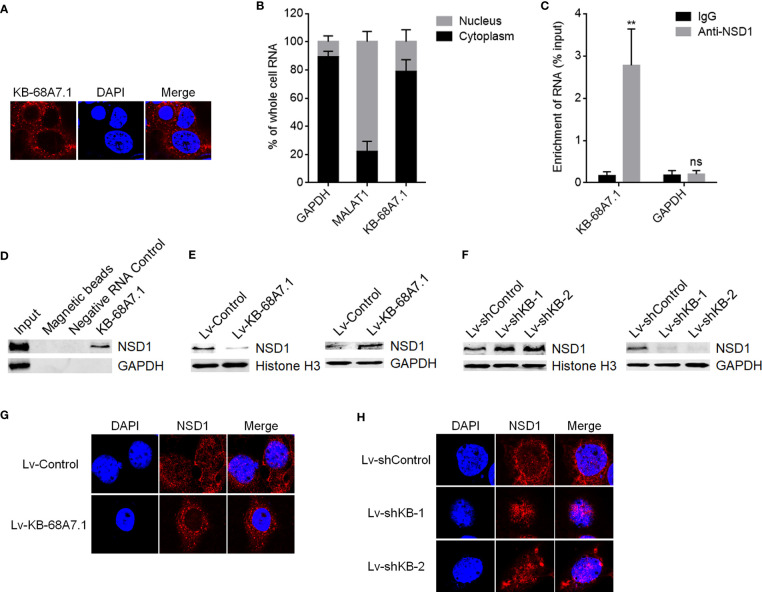
KB-68A7.1 bound to NSD1 and redistributed NSD1 to the cytoplasm. **(A)** Subcellular distribution of KB-68A7.1 in SNU-398 cells was assessed by RNA FISH. **(B)** Subcellular distribution of KB-68A7.1 in SNU-398 cells was assessed by cytoplasmic and nuclear RNA purification, followed by RT-qPCR. GAPDH and MALAT1 were used as cytoplasmic and nuclear RNA controls, respectively. **(C)** RIP assays were undertaken in SNU-398 cells using NSD1 primary antibody or non-specific IgG. Enrichment of RNA was assessed by RT-qPCR. **(D)** RNA–protein pull-down assay were undertaken in SNU-398 cells using RNA end-labeled with desthiobiotin. Enrichment of protein was assessed by Western blot. **(E)** Nuclear and cytoplasmic NSD1 levels in SNU-398 cells with KB-68A7.1 overexpression or control was measured by Western blot. Histone H3 and GAPDH were used as loading controls for nuclear and cytoplasmic proteins, respectively. **(F)** Nuclear and cytoplasmic NSD1 levels in SNU-398 cells with KB-68A7.1 silencing or control was measured by Western blot. **(G)** Subcellular distribution of NSD1 in SNU-398 cells with KB-68A7.1 overexpression or control was assessed by IF. **(H)** Subcellular distribution of NSD1 in SNU-398 cells with KB-68A7.1 silencing or control was assessed by IF. Results are shown as mean ± SD based on three independent experiments. **p < 0.01, ns, not significant by two-tailed unpaired t-test.

To explore the mechanisms mediating the roles of KB-68A7.1 in HCC, we first assessed the subcellular distribution of KB-68A7.1 in SNU-398 cells using RNA fluorescence *in situ* hybridization (FISH), which demonstrated that KB-68A7.1 was mostly distributed in the cytoplasm (**Figure 5A**). Cytoplasmic and nuclear RNA purification, followed by RT-qPCR once again showed the cytoplasmic distribution of KB-68A7.1 in SNU-398 cells (**Figure 5B**). One of the important mechanisms of cytoplasmic lncRNAs is to bind proteins. Thus, the online *in silico* tool RNA-Protein Interaction Prediction (RPISeq) (http://pridb.gdcb.iastate.edu/RPISeq/) was used to predict the proteins bound by KB-68A7.1. Intriguingly, histone lysine methyltransferase NSD1 was predicted to interact with KB-68A7.1 with RF score of 0.85 and SVM score of 0.9. RIP assay revealed that KB-68A7.1 was specifically enriched by NSD1 primary antibody (**Figure 5C**). RNA–protein pull-down assay using RNA end-labeled with desthiobiotin revealed that NSD1 was specifically enriched by KB-68A7.1 (**Figure 5D**). Therefore, the RIP assay and RNA–protein pull-down assay both confirmed the interaction between KB-68A7.1 and NSD1. Considering the cytoplasmic location of KB-68A7.1, we next investigated whether KB-68A7.1 regulates nuclear distribution of NSD1. Nuclear and cytoplasmic proteins were isolated from SNU-398 cells with KB-68A7.1 stable overexpression or depletion, followed by Western blot to assess nuclear and cytoplasmic NSD1 levels. The results found that ectopic expression of KB-68A7.1 downregulated nuclear NSD1 level and increased cytoplasmic NSD1 level (**Figure 5E**). Silencing of KB-68A7.1 increased nuclear NSD1 level and reduced cytoplasmic NSD1 level (**Figure 5F**). IF assays once again confirmed that SNU-398 cells with KB-68A7.1 overexpression had reduced nuclear NSD1 level, and SNU-398 cells with KB-68A7.1 depletion had increased nuclear NSD1 level (**Figures 5G, H**). Taken together, these results demonstrated that cytoplasmic KB-68A7.1 bound to NSD1 and reduced the nuclear distribution of NSD1.

### KB-68A7.1 Repressed *WNT10B* Transcription Through Restricting NSD1

**Figure 6:**
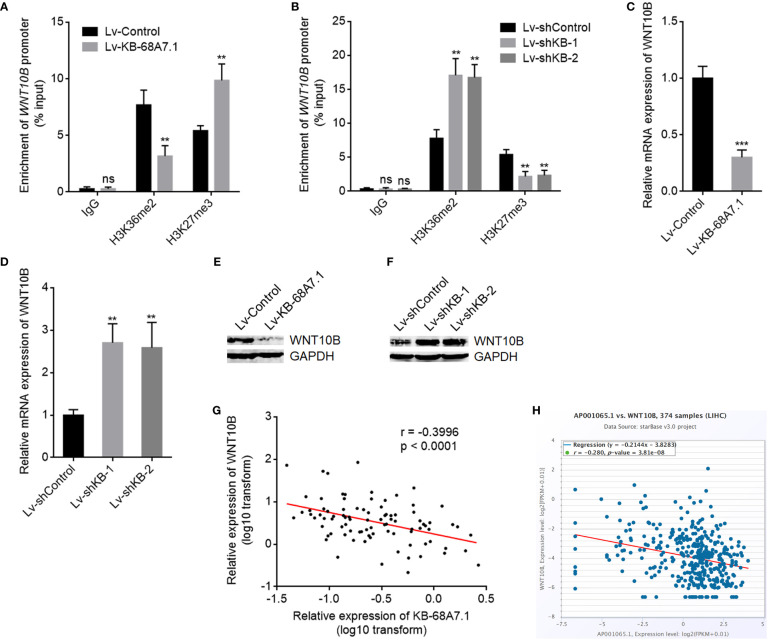
KB-68A7.1 repressed *WNT10B* transcription. **(A)** ChIP assays were undertaken in SNU-398 cells with KB-68A7.1 overexpression or control using H3K36me2, H3K27me3 primary antibodies, or non-specific IgG. Enrichment of *WNT10B* promoter was assessed by qPCR. **(B)** ChIP assays were undertaken in SNU-398 cells with KB-68A7.1 silencing or control using H3K36me2, H3K27me3 primary antibodies, or non-specific IgG. Enrichment of *WNT10B* promoter was assessed by qPCR. **(C)** WNT10B mRNA expression in SNU-398 cells with KB-68A7.1 overexpression or control was measured by RT-qPCR. **(D)** WNT10B mRNA expression in SNU-398 cells with KB-68A7.1 silencing or control was measured by RT-qPCR. **(E)** WNT10B protein level in SNU-398 cells with KB-68A7.1 overexpression or control was measured by Western blot. **(F)** WNT10B protein level in SNU-398 cells with KB-68A7.1 silencing or control was measured by Western blot. **(G)** The correlation between WNT10B and KB-68A7.1 expression in these 90 HCC tissues. r = −0.3996, p < 0.0001 by Spearman correlation analysis. **(H)** The correlation between WNT10B and KB-68A7.1 (AP001065.1) expression in HCC tissues according to TCGA LIHC dataset was analysed by the online *in silico* tool ENCORI. For Panels **(A–D)**, results are shown as mean ± SD based on three independent experiments. **p < 0.01, ***p < 0.001, ns, not significant by two-tailed unpaired t-test **(A, C)** or one-way ANOVA followed by Dunnett’s multiple comparisons test **(B, D)**.

Our previous study has found that NSD1 induced H3K36me2 at *WNT10B* promoter and further repressed H3K27me3 at *WNT10B* promoter, leading to transcription activation of *WNT10B* (19). Therefore, we next investigated whether KB-68A7.1 modulates *WNT10B*. ChIP assays were undertaken in SNU-398 cells with KB-68A7.1 stable overexpression or depletion to assess H3K36me2 and H3K27me3 levels at *WNT10B* promoter, which found that ectopic expression of KB-68A7.1 reduced H3K36me2 level and increased H3K27me3 level at *WNT10B* promoter (**Figure 6A**), while silencing of KB-68A7.1 increased H3K36me2 level and reduced H3K27me3 level at *WNT10B* promoter (**Figure 6B**). Consistent with the changes in histone methylation, *WNT10B* transcription was decreased in SNU-398 cells with KB-68A7.1 overexpression and increased in SNU-398 cells with KB-68A7.1 depletion (**Figures 6C, D**). WNT10B protein level was consistently decreased in SNU-398 cells with KB-68A7.1 overexpression and increased in SNU-398 cells with KB-68A7.1 depletion (**Figures 6E, F**). Ectopic expression of KB-68A7.1 also reduced WNT10B expression in HuH-7 cells (**Supplementary Figure S2A**). Silencing of KB-68A7.1 also increased WNT10B expression in SK-HEP-1 and THLE-3 cells (**Supplementary Figures S2B, C**). To investigate the relevance between KB-68A7.1 and WNT10B *in vivo*, WNT10B expression was measured in the same HCC tissues used in **Figure 1C**, and the result presented that the expression of WNT10B was inversely correlated with that of KB-68A7.1 in HCC tissues (**Figure 6G**). The inverse correlation between WNT10B and KB-68A7.1 expression was further found in TCGA LIHC dataset (**Figure 6H**), analysed by the online *in silico* tool ENCORI (https://starbase.sysu.edu.cn/) (45). Taken together, these results demonstrated that conversely with NSD1, KB-68A7.1 repressed *WNT10B* transcription.

### KB-68A7.1 Repressed Wnt/β-Catenin Signalling

**Figure 7:**
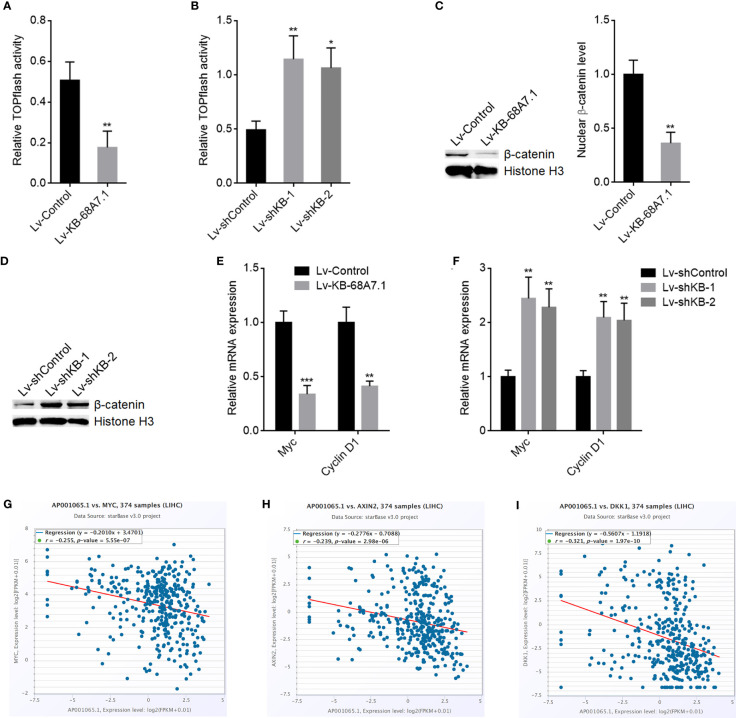
KB-68A7.1 repressed Wnt/β-catenin signalling. **(A)** Wnt/β-catenin reporter TOPflash was co-transfected with pRL-TK into SNU-398 cells with KB-68A7.1 overexpression or control. Luciferase activities were measured 48 h after transfection. Results were shown as the ratio of Firefly luciferase activity to Renilla luciferase activity. **(B)** Wnt/β-catenin reporter TOPflash was co-transfected with pRL-TK into SNU-398 cells with KB-68A7.1 silencing or control. Luciferase activities were measured 48 h after transfection. Results were shown as the ratio of Firefly luciferase activity to Renilla luciferase activity. **(C)** Nuclear β-catenin level in SNU-398 cells with KB-68A7.1 overexpression or control was measured by Western blot. **(D)** Nuclear β-catenin level in SNU-398 cells with KB-68A7.1 silencing or control was measured by Western blot. **(E)** Myc and cyclin D1 expressions in SNU-398 cells with KB-68A7.1 overexpression or control were measured by RT-qPCR. **(F)** Myc and Cyclin D1 expressions in SNU-398 cells with KB-68A7.1 silencing or control was measured by RT-qPCR. **(G–I)** The correlations between MYC **(G)**, AXIN2 **(H)**, DKK1 **(I)**, and KB-68A7.1 (AP001065.1) expression in HCC tissues according to TCGA LIHC dataset were analysed by the online *in silico* tool ENCORI. For Panels **(A**–**F)**, results are shown as mean ± SD based on three independent experiments. *p < 0.05, **p < 0.01, ***p < 0.001 by two-tailed unpaired t-test **(A, C, E)** or one-way ANOVA followed by Dunnett’s multiple comparisons test **(B, F)**.

WNT10B is well known to activate Wnt/β-catenin signalling (46, 47). Therefore, we further explored the potential effects of KB-68A7.1 on Wnt/β-catenin signalling. Wnt/β-catenin reporter TOPflash luciferase activities were assessed in SNU-398 cells with KB-68A7.1 stable overexpression or depletion, which presented that ectopic expression of KB-68A7.1 reduced TOPflash luciferase activity, while silencing of KB-68A7.1 increased TOPflash luciferase activity (**Figures 7A, B**). Ectopic expression of KB-68A7.1 also reduced TOPflash luciferase activity in HuH-7 cells (**Supplementary Figure S2D**). Silencing of KB-68A7.1 also increased TOPflash luciferase activity in SK-HEP-1 and THLE-3 cells (**Supplementary Figures S2E, F**). Nuclear β-catenin level was decreased in SNU-398 cells with KB-68A7.1 overexpression and increased in SNU-398 cells with KB-68A7.1 depletion (**Figures 7C, D**). Furthermore, Wnt/β-catenin signalling downstream targets Myc and Cyclin D1 were decreased in SNU-398 cells with KB-68A7.1 overexpression and increased in SNU-398 cells with KB-68A7.1 depletion (**Figures 7E, F**). Ectopic expression of KB-68A7.1 also reduced Myc and Cyclin D1 expression in HuH-7 cells (**Supplementary Figure S2G**). Silencing of KB-68A7.1 also increased Myc and cyclin D1 expression in SK-HEP-1 and THLE-3 cells (**Supplementary Figures S2H, I**). The expression of KB-68A7.1 was inversely correlated with that of Wnt/β-catenin targets MYC, AXIN2, and DKK1 in HCC tissues, according to TCGA LIHC dataset (**Figures 7G–I**), supporting the clinical relevance between KB-68A7.1 and Wnt/β-catenin signalling *in vivo*. Taken together, these data demonstrated that consistent with the repression of *WNT10B* transcription, Wnt/β-catenin signalling was also repressed by KB-68A7.1.

### The Tumour Suppressive Roles of KB-68A7.1 in HCC Were Dependent on the WNT10B/Wnt/β-Catenin Signalling Axis

**Figure 8:**
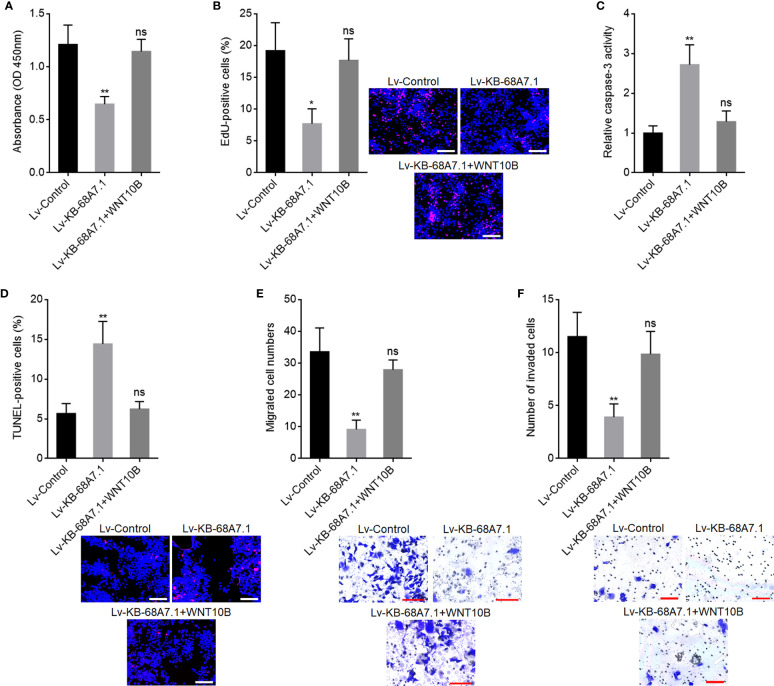
WNT10B reversed the tumour-suppressive roles of KB-68A7.1 in HCC. **(A)** Cellular proliferation of SNU-398 cells with KB-68A7.1 and WNT10B overexpression or control was measured by CCK-8 assay. **(B)** Cellular proliferation of SNU-398 cells with KB-68A7.1 and WNT10B overexpression or control was measured by EdU incorporation assay. Red colour indicates EdU-positive and proliferative cells. Scale bars = 100 µm. **(C)** Cellular apoptosis of SNU-398 cells with KB-68A7.1 and WNT10B overexpression or control was measured by caspase-3 activity assay. **(D)** Cellular apoptosis of SNU-398 cells with KB-68A7.1 and WNT10B overexpression or control was measured by TUNEL assay. Red colour indicates TUNEL-positive and apoptotic cells. Scale bars = 100 µm. **(E)** Cellular migration of SNU-398 cells with KB-68A7.1 and WNT10B overexpression or control was measured by Transwell migration assay. Scale bars = 100 µm. **(F)** Cell invasion of SNU-398 cells with KB-68A7.1 and WNT10B overexpression or control was measured by Transwell invasion assay. Scale bars = 100 µm. Results are shown as mean ± SD based on three independent experiments. *p < 0.05, **p < 0.01, ns, not significant by one-way ANOVA followed by Dunnett’s multiple comparisons test.

**Figure 9:**
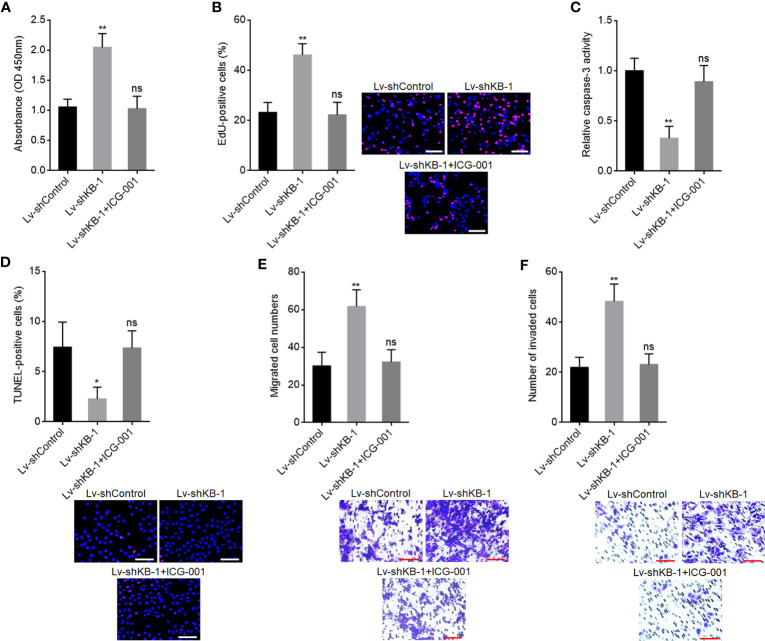
Wnt/β-catenin signalling inhibitor ICG-001 reversed the oncogenic roles of KB-68A7.1 depletion in HCC. **(A)** Cellular proliferation of KB-68A7.1 silenced SK-HEP-1 cells treated with 5 μM ICG-001 was measured by CCK-8 assay. **(B)** Cellular proliferation of KB-68A7.1 silenced SK-HEP-1 cells treated with 5 μM ICG-001 was measured by EdU incorporation assay. Red colour indicates EdU-positive and proliferative cells. Scale bars = 100 µm. **(C)** Cellular apoptosis of KB-68A7.1-silenced SK-HEP-1 cells treated with 5 μM ICG-001 was measured by caspase-3 activity assay. **(D)** Cellular apoptosis of KB-68A7.1-silenced SK-HEP-1 cells treated with 5 μM ICG-001 was measured by TUNEL assay. Red colour indicates TUNEL-positive and apoptotic cells. Scale bars = 100 µm. **(E)** Cellular migration of KB-68A7.1-silenced SK-HEP-1 cells treated with 5 μM ICG-001 was measured by Transwell migration assay. Scale bars = 100 µm. **(F)** Cell invasion of KB-68A7.1-silenced SK-HEP-1 cells treated with 5 μM ICG-001 measured by Transwell invasion assay. Scale bars = 100 µm. Results are shown as mean ± SD based on three independent experiments. *p < 0.05, **p < 0.01, ns, not significant by one-way ANOVA followed by Dunnett’s multiple comparisons test.

To explore whether the roles of KB-68A7.1 in HCC were dependent on WNT10B, WNT10B was stably overexpressed in SNU-398 cells with KB-68A7.1 stable overexpression. CCK-8 and EdU incorporation assays demonstrated that ectopic expression of WNT10B reversed the reduced cellular proliferation ability caused by KB-68A7.1 overexpression (**Figures 8A, B**). Caspase-3 activity assay and TUNEL assay demonstrated that ectopic expression of WNT10B reversed the induced cellular apoptosis caused by KB-68A7.1 overexpression (**Figures 8C, D**). Transwell migration and invasion assays demonstrated that ectopic expression of WNT10B reversed the reduced cellular migration and invasion caused by KB-68A7.1 overexpression (**Figures 8E, F**). Moreover, WNT10B was also overexpressed in HuH-7 cells, CCK-8 assay, EdU incorporation assay, caspase-3 activity assay, TUNEL assay, and Transwell migration and invasion assays, all demonstrated that ectopic expression of WNT10B partially reversed the tumour-suppressive roles of KB-68A7.1 in HuH-7 cells (**Supplementary Figure S3**). To further explore whether the roles of KB-68A7.1 in HCC were dependent on Wnt/β-catenin signalling, SK-HEP-1 cells with KB-68A7.1 stable depletion were treated with Wnt/β-catenin signalling inhibitor ICG-001. CCK-8 and EdU incorporation assays demonstrated that treatment with ICG-001 reversed the increased cellular proliferation ability caused by KB-68A7.1 depletion (**Figures 9A, B**). Caspase-3 activity assay and TUNEL assay demonstrated that treatment with ICG-001 reversed the reduced cellular apoptosis caused by KB-68A7.1 depletion (**Figures 9C, D**). Transwell migration and invasion assays demonstrated that treatment with ICG-001 reversed the increased cellular migration and invasion caused by KB-68A7.1 depletion (**Figures 9E, F**). Taken together, these results demonstrated that the roles of KB-68A7.1 in HCC were dependent on the WNT10B/Wnt/β-catenin signalling axis.

## Discussion

HCC is one of the most lethal malignancies, only next to pancreatic cancer (3). HCC has quick progression and therefore poor prognosis (48). Revealing the critical molecules driving HCC development is of clinical importance. In this study, we identified a novel HCC-related lncRNA KB-68A7.1, which represses HCC development. Both public dataset and our own cohort found that KB-68A7.1 was lowly expressed in HCC tissues compared with liver tissues, and its low expression was correlated with poor overall survival of HCC patients. Except KB-68A7.1, other lncRNAs were also previously reported to be correlated with prognosis of HCC patients, such as lncRNA-ATB, PVT1, CASC9, and GPC3-AS1 (37, 49–51). The combination of these lncRNAs would be more efficient for predicting HCCs’ prognosis, which need multi-central investigations. The expression pattern and clinical relevance of KB-68A7.1 in other malignancies have not been studied, which also need further investigation to confirm whether KB-68A7.1 is HCC specific or cancer popular.

Functional studies revealed KB-68A7.1 as a tumour suppressor in HCC. Ectopic expression of KB-68A7.1 restricted HCC cellular proliferation and induced HCC cellular apoptosis *in vitro* and *in vivo*. Thus, KB-68A7.1 inhibited HCC tumour growth *in vivo*. Furthermore, ectopic expression of KB-68A7.1 inhibited HCC cellular migration and invasion *in vitro* and further inhibited HCC metastasis *in vivo*. These findings suggested that ectopic expression of KB-68A7.1 or inducing KB-68A7.1 expression would be potential strategy for HCC therapy.

There are various mechanisms of action of lncRNAs (39). Routinely, nuclear lncRNAs bind epigenetic modification complex, change genomic localization of the complex, and therefore modulate the epigenetic modification and transcription of target genes (37). Cytoplasmic lncRNAs could bind microRNAs, relieve the repressive roles of microRNAs on their targets, and therefore increase the expressions of these targets (49). Cytoplasmic lncRNAs could also directly bind mRNAs and regulate their stability and/or translation (52). Furthermore, cytoplasmic lncRNAs may bind proteins and modulate the stability and/or function of the interacted proteins (53). Here, we first confirmed that KB-68A7.1 was mainly distributed in the cytoplasm. Intriguingly, here we found that cytoplasmic KB-68A7.1 directly bound to NSD1. NSD1 is a histone lysine methyltransferase, which exerts function in nucleus (20). The binding of KB-68A7.1 to NSD1 sequestrated NSD1 in the cytoplasm, leading to the reduction in nuclear NSD1 level. Thus, the binding of KB-68A7.1 to NSD1 also attenuated the functions of NSD1. As a chromatin modifier, NSD1 has various roles in different pathophysiological processes (54, 55). Through catalyzing H3K36me2, NSD1 further modulates H3K27me3, DNA methylation, and/or acetylation of histone H3 at lysine 27 (H3K27ac), leading to activation or suppression of gene expression (20, 54). In HCC, we have previously identified WNT10B as a critical downstream target of NSD1, which mediated the oncogenic roles of NSD1 in HCC (19). In this study, we further found that through binding to and sequestrating NSD1 in the cytoplasm, KB-68A7.1 repressed NSD1-induced H3K36me2 increasing and H3K27me3 reduction at *WNT10B* promoter, leading to the repression of NSD1-induced *WNT10B* transcription activation. Therefore, KB-68A7.1 repressed WNT10B expression in HCC. WNT10B is a classical Wnt/β-catenin signalling activator (46). Wnt/β-catenin signalling activation contributes to malignant progression of many cancers, including HCC (56). Here, we found that through reducing WNT10B expression, KB-68A7.1 further repressed Wnt/β-catenin signalling. Functional rescue experiments found that WNT10B/Wnt/β-catenin signalling axis are critical mediators of the roles of KB-68A7.1 in HCC. Through regulating NSD1, KB-68A7.1 may influence epigenetic modifications of other target genes, which may also mediate the roles of KB-68A7.1 in HCC. The potential effects of KB-68A7.1 on other targets need further explorations.

In conclusion, these data demonstrated that KB-68A7.1 is lowly expressed in HCC and its low expression is correlated with poor survival of HCC patients. KB-68A7.1 functions as a tumour suppressor *via* binding to and sequestrating NSD1 in the cytoplasm, reducing *WNT10B* transcription, and repressing Wnt/β-catenin signalling. This study suggested KB-68A7.1 as a potential target for HCC prognostic prediction and targeted therapy.

## Data Availability Statement

The original contributions presented in the study are included in the article/**Supplementary Material**. Further inquiries can be directed to the corresponding author.

## Ethics Statement

The studies involving human participants were reviewed and approved by the Ethic Committee of Union Hospital, Tongji Medical College, Huazhong University of Science and Technology. The patients/participants provided their written informed consent to participate in this study. The animal study was reviewed and approved by the Institutional Animal Care and Use Committee of Union Hospital, Tongji Medical College, Huazhong University of Science and Technology.

## Author Contributions

SZ and JXu conceived this study. SZ, JXu, HC, and MJ conducted the experiments. SZ, JXu, HC, and JXi analysed the data. SZ and JXu wrote the manuscript. All authors contributed to the article and approved the submitted version.

## Funding

This work was supported by the Nature Science Foundation of Hubei Province, China (2020CFB796).

## Conflict of Interest

The authors declare that the research was conducted in the absence of any commercial or financial relationships that could be construed as a potential conflict of interest.

## Publisher’s Note

All claims expressed in this article are solely those of the authors and do not necessarily represent those of their affiliated organizations, or those of the publisher, the editors and the reviewers. Any product that may be evaluated in this article, or claim that may be made by its manufacturer, is not guaranteed or endorsed by the publisher.

## Supplementary Material

Supplementary Figure 1 The roles of KB-68A7.1 silencing in THLE-3 cellular proliferation, apoptosis, migration, and invasion. **(A)** KB-68A7.1 expression in THLE-3 cells with KB-68A7.1 silencing or control was measured by RT-qPCR. **(B)** Cellular proliferation of THLE-3 cells with KB-68A7.1 silencing or control was measured by CCK-8 assay. **(C)** Cellular proliferation of THLE-3 cells with KB-68A7.1 silencing or control was measured by EdU incorporation assay. Representative images are shown on the right. Red color indicates EdU-positive and proliferative cells. Scale bars = 100 µm. **(D)** Cellular apoptosis of THLE-3 cells with KB-68A7.1 silencing or control was measured by caspase-3 activity assay. **(E)** Cellular apoptosis of THLE-3 cells with KB-68A7.1 silencing or control was measured by TUNEL assay. Representative images are shown on the right. Red color indicates TUNEL-positive and apoptotic cells. Scale bars = 100 µm. **(F)** Cellular migration of THLE-3 cells with KB-68A7.1 silencing or control was measured by transwell migration assay. Representative images are shown on the right. Scale bars = 100 µm. **(G)** Cell invasion of THLE-3 cells with KB-68A7.1 silencing or control was measured by transwell invasion assay. Representative images are shown on the right. Scale bars = 100 µm. Results are shown as mean ± SD based on three independent experiments. *p < 0.05, **p < 0.01, ***p < 0.001 by two-tailed unpaired t test.

Supplementary Figure 2 KB-68A7.1 repressed WNT10B expression and Wnt/β-catenin signaling. **(A)** WNT10B expression in HuH-7 cells with KB-68A7.1 overexpression or control was measured by RT-qPCR. **(B)** WNT10B expression in SK-HEP-1 cells with KB-68A7.1 silencing or control was measured by RT-qPCR. **(C)** WNT10B expression in THLE-3 cells with KB-68A7.1 silencing or control was measured by RT-qPCR. **(D)** Wnt/β-catenin reporter TOPflash was co-transfected with pRL-TK into HuH-7 cells with KB-68A7.1 overexpression or control. Luciferase activities were measured 48h after transfection. Results were shown as the ratio of Firefly luciferase activity to Renilla luciferase activity. **(E)** Wnt/β-catenin reporter TOPflash was co-transfected with pRL-TK into SK-HEP-1 cells with KB-68A7.1 silencing or control. Luciferase activities were measured 48h after transfection. Results were shown as the ratio of Firefly luciferase activity to Renilla luciferase activity. **(F)** Wnt/β-catenin reporter TOPflash was co-transfected with pRL-TK into THLE-3 cells with KB-68A7.1 silencing or control. Luciferase activities were measured 48h after transfection. Results were shown as the ratio of Firefly luciferase activity to Renilla luciferase activity. **(G)** Myc and Cyclin D1 expressions in HuH-7 cells with KB-68A7.1 overexpression or control were measured by RT-qPCR. **(H)** Myc and Cyclin D1 expressions in SK-HEP-1 cells with KB-68A7.1 silencing or control was measured by RT-qPCR. **(I)** Myc and Cyclin D1 expressions in THLE-3 cells with KB-68A7.1 silencing or control was measured by RT-qPCR. Results are shown as mean ± SD based on three independent experiments. *p < 0.05, **p < 0.01 by two-tailed unpaired t test.

Supplementary Figure 3 WNT10B partially reversed the tumor suppressive roles of KB-68A7.1 in HuH-7 cells. **(A)** Cellular proliferation of HuH-7 cells with KB-68A7.1 and WNT10B overexpression or control was measured by CCK-8 assay. **(B)** Cellular proliferation of HuH-7 cells with KB-68A7.1 and WNT10B overexpression or control was measured by EdU incorporation assay. Red color indicates EdU-positive and proliferative cells. Scale bars = 100 µm. **(C)** Cellular apoptosis of HuH-7 cells with KB-68A7.1 and WNT10B overexpression or control was measured by caspase-3 activity assay. **(D)** Cellular apoptosis of HuH-7 cells with KB-68A7.1 and WNT10B overexpression or control was measured by TUNEL assay. Red color indicates TUNEL-positive and apoptotic cells. Scale bars = 100 µm. **(E)** Cellular migration of HuH-7 cells with KB-68A7.1 and WNT10B overexpression or control was measured by transwell migration assay. Scale bars = 100 µm. **(F)** Cell invasion of HuH-7 cells with KB-68A7.1 and WNT10B overexpression or control was measured by transwell invasion assay. Scale bars = 100 µm. Results are shown as mean ± SD based on three independent experiments. *p < 0.05, **p < 0.01, ns, not significant by one-way ANOVA followed by Dunnett's multiple comparisons test.
