# Supplementary figures and images for: KB-68A7.1 Inhibits Hepatocellular Carcinoma Development Through Binding to NSD1 and Suppressing Wnt/β-Catenin Signalling
Source: Front Oncol. 2022 Jan 20;11:808291. doi: 10.3389/fonc.2021.808291 (PMC8810504; doi:10.3389/fonc.2021.808291)

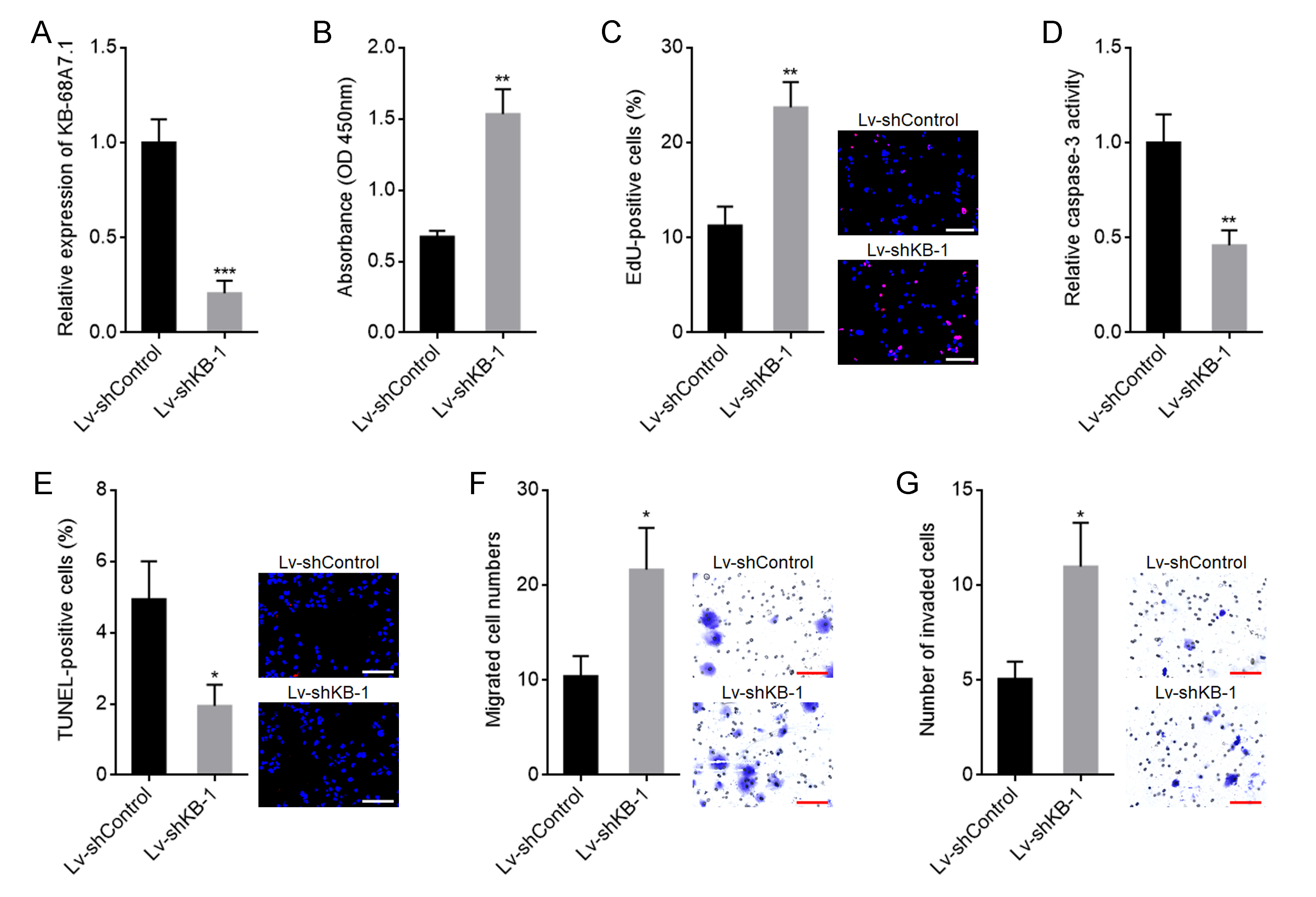

Supplement: Supplementary Figure 1 — The roles of KB-68A7.1 silencing in THLE-3 cellular proliferation, apoptosis, migration, and invasion. (A) KB-68A7.1 expression in THLE-3 cells with KB-68A7.1 silencing or control was measured by RT-qPCR. (B) Cellular proliferation of THLE-3 cells with KB-68A7.1 silencing or control was measured by CCK-8 assay. (C) Cellular proliferation of THLE-3 cells with KB-68A7.1 silencing or control was measured by EdU incorporation assay. Representative images are shown on the right. Red color indicates EdU-positive and proliferative cells. Scale bars = 100 µm. (D) Cellular apoptosis of THLE-3 cells with KB-68A7.1 silencing or control was measured by caspase-3 activity assay. (E) Cellular apoptosis of THLE-3 cells with KB-68A7.1 silencing or control was measured by TUNEL assay. Representative images are shown on the right. Red color indicates TUNEL-positive and apoptotic cells. Scale bars = 100 µm. (F) Cellular migration of THLE-3 cells with KB-68A7.1 silencing or control was measured by transwell migration assay. Representative images are shown on the right. Scale bars = 100 µm. (G) Cell invasion of THLE-3 cells with KB-68A7.1 silencing or control was measured by transwell invasion assay. Representative images are shown on the right. Scale bars = 100 µm. Results are shown as mean ± SD based on three independent experiments. *p < 0.05, **p < 0.01, ***p < 0.001 by two-tailed unpaired t test. [file Image_1.tif]

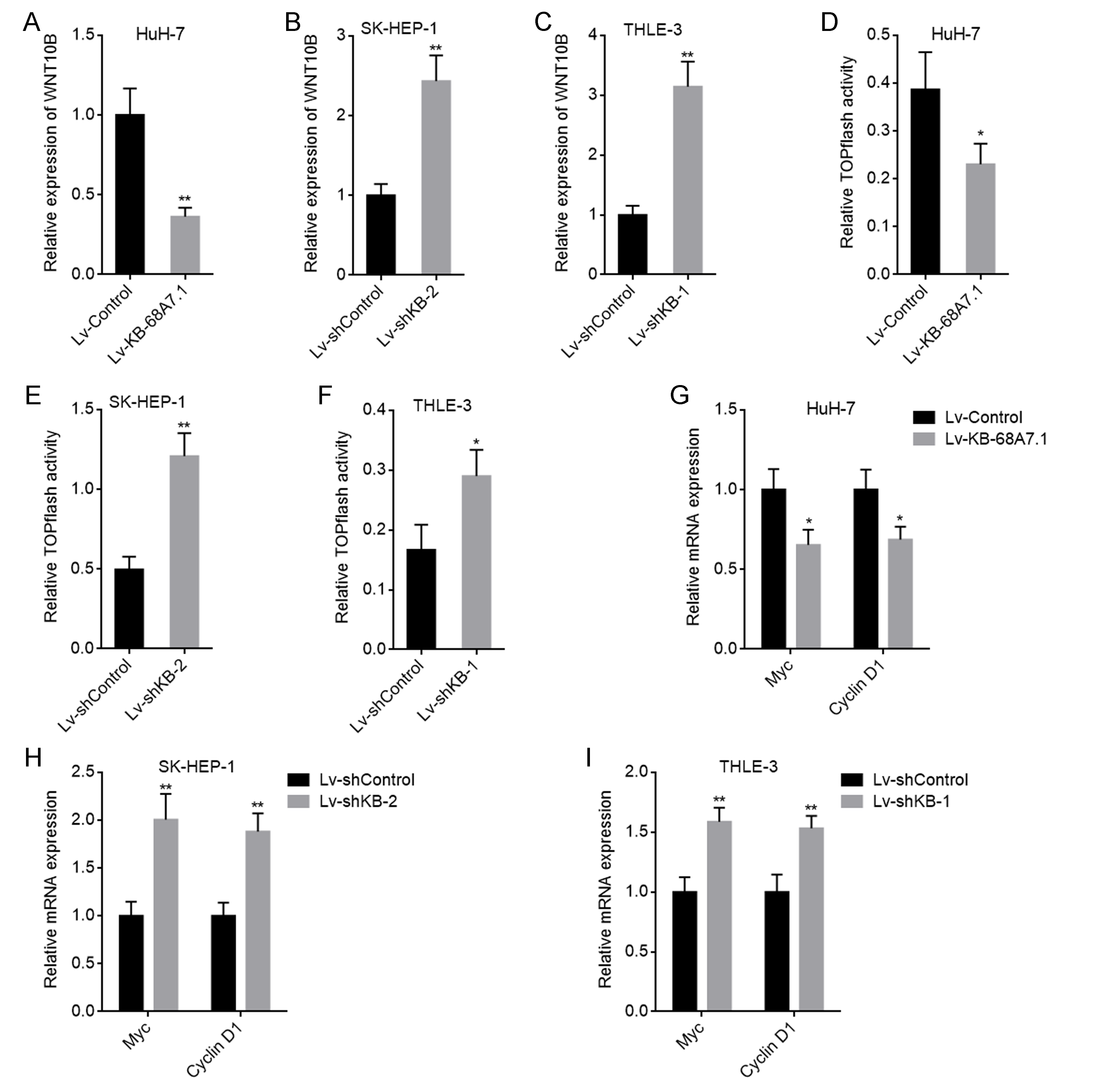

Supplement: Supplementary Figure 2 — KB-68A7.1 repressed WNT10B expression and Wnt/β-catenin signaling. (A) WNT10B expression in HuH-7 cells with KB-68A7.1 overexpression or control was measured by RT-qPCR. (B) WNT10B expression in SK-HEP-1 cells with KB-68A7.1 silencing or control was measured by RT-qPCR. (C) WNT10B expression in THLE-3 cells with KB-68A7.1 silencing or control was measured by RT-qPCR. (D) Wnt/β-catenin reporter TOPflash was co-transfected with pRL-TK into HuH-7 cells with KB-68A7.1 overexpression or control. Luciferase activities were measured 48h after transfection. Results were shown as the ratio of Firefly luciferase activity to Renilla luciferase activity. (E) Wnt/β-catenin reporter TOPflash was co-transfected with pRL-TK into SK-HEP-1 cells with KB-68A7.1 silencing or control. Luciferase activities were measured 48h after transfection. Results were shown as the ratio of Firefly luciferase activity to Renilla luciferase activity. (F) Wnt/β-catenin reporter TOPflash was co-transfected with pRL-TK into THLE-3 cells with KB-68A7.1 silencing or control. Luciferase activities were measured 48h after transfection. Results were shown as the ratio of Firefly luciferase activity to Renilla luciferase activity. (G) Myc and Cyclin D1 expressions in HuH-7 cells with KB-68A7.1 overexpression or control were measured by RT-qPCR. (H) Myc and Cyclin D1 expressions in SK-HEP-1 cells with KB-68A7.1 silencing or control was measured by RT-qPCR. (I) Myc and Cyclin D1 expressions in THLE-3 cells with KB-68A7.1 silencing or control was measured by RT-qPCR. Results are shown as mean ± SD based on three independent experiments. *p < 0.05, **p < 0.01 by two-tailed unpaired t test. [file Image_2.tif]

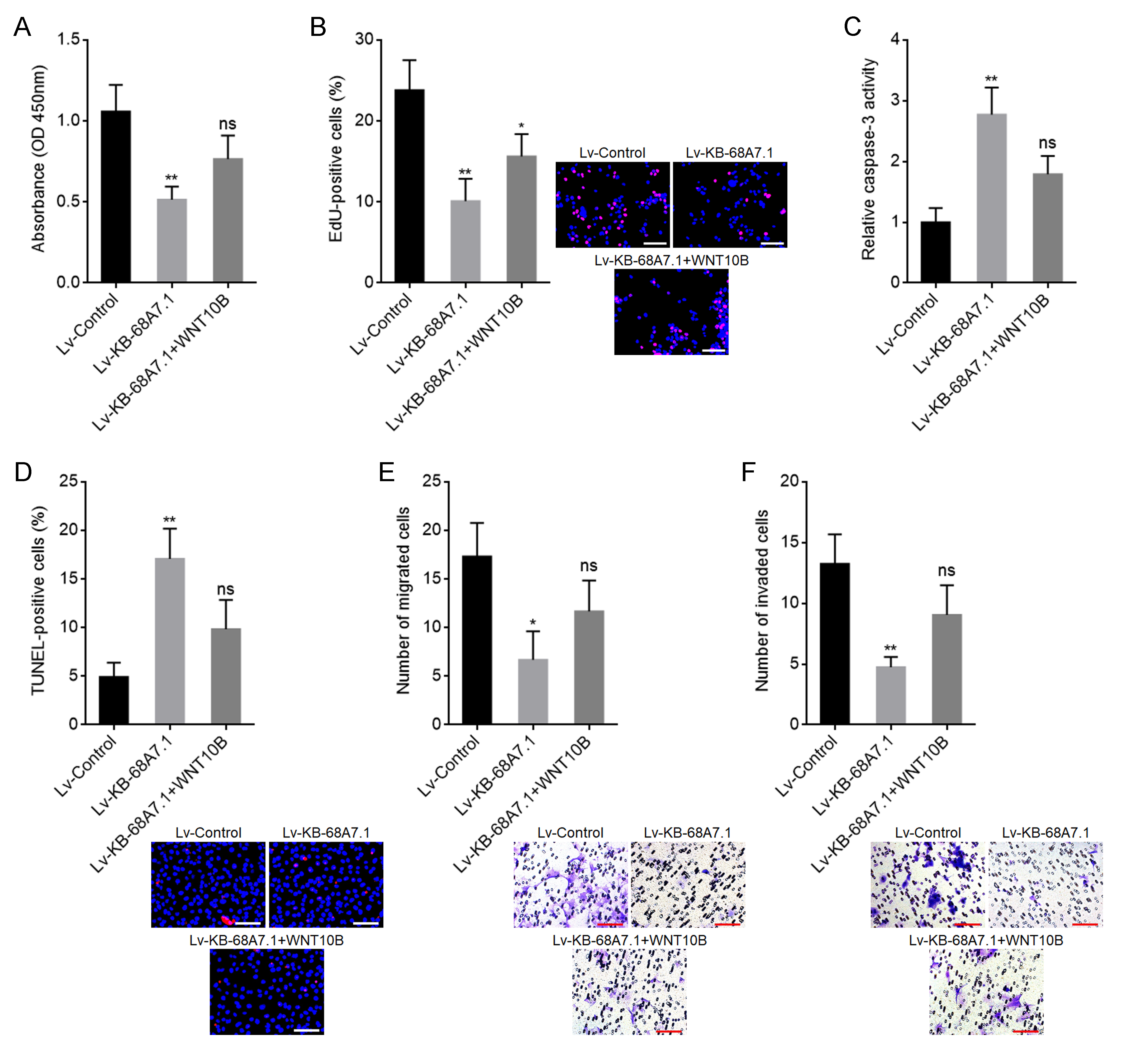

Supplement: Supplementary Figure 3 — WNT10B partially reversed the tumor suppressive roles of KB-68A7.1 in HuH-7 cells. (A) Cellular proliferation of HuH-7 cells with KB-68A7.1 and WNT10B overexpression or control was measured by CCK-8 assay. (B) Cellular proliferation of HuH-7 cells with KB-68A7.1 and WNT10B overexpression or control was measured by EdU incorporation assay. Red color indicates EdU-positive and proliferative cells. Scale bars = 100 µm. (C) Cellular apoptosis of HuH-7 cells with KB-68A7.1 and WNT10B overexpression or control was measured by caspase-3 activity assay. (D) Cellular apoptosis of HuH-7 cells with KB-68A7.1 and WNT10B overexpression or control was measured by TUNEL assay. Red color indicates TUNEL-positive and apoptotic cells. Scale bars = 100 µm. (E) Cellular migration of HuH-7 cells with KB-68A7.1 and WNT10B overexpression or control was measured by transwell migration assay. Scale bars = 100 µm. (F) Cell invasion of HuH-7 cells with KB-68A7.1 and WNT10B overexpression or control was measured by transwell invasion assay. Scale bars = 100 µm. Results are shown as mean ± SD based on three independent experiments. *p < 0.05, **p < 0.01, ns, not significant by one-way ANOVA followed by Dunnett's multiple comparisons test. [file Image_3.tif]

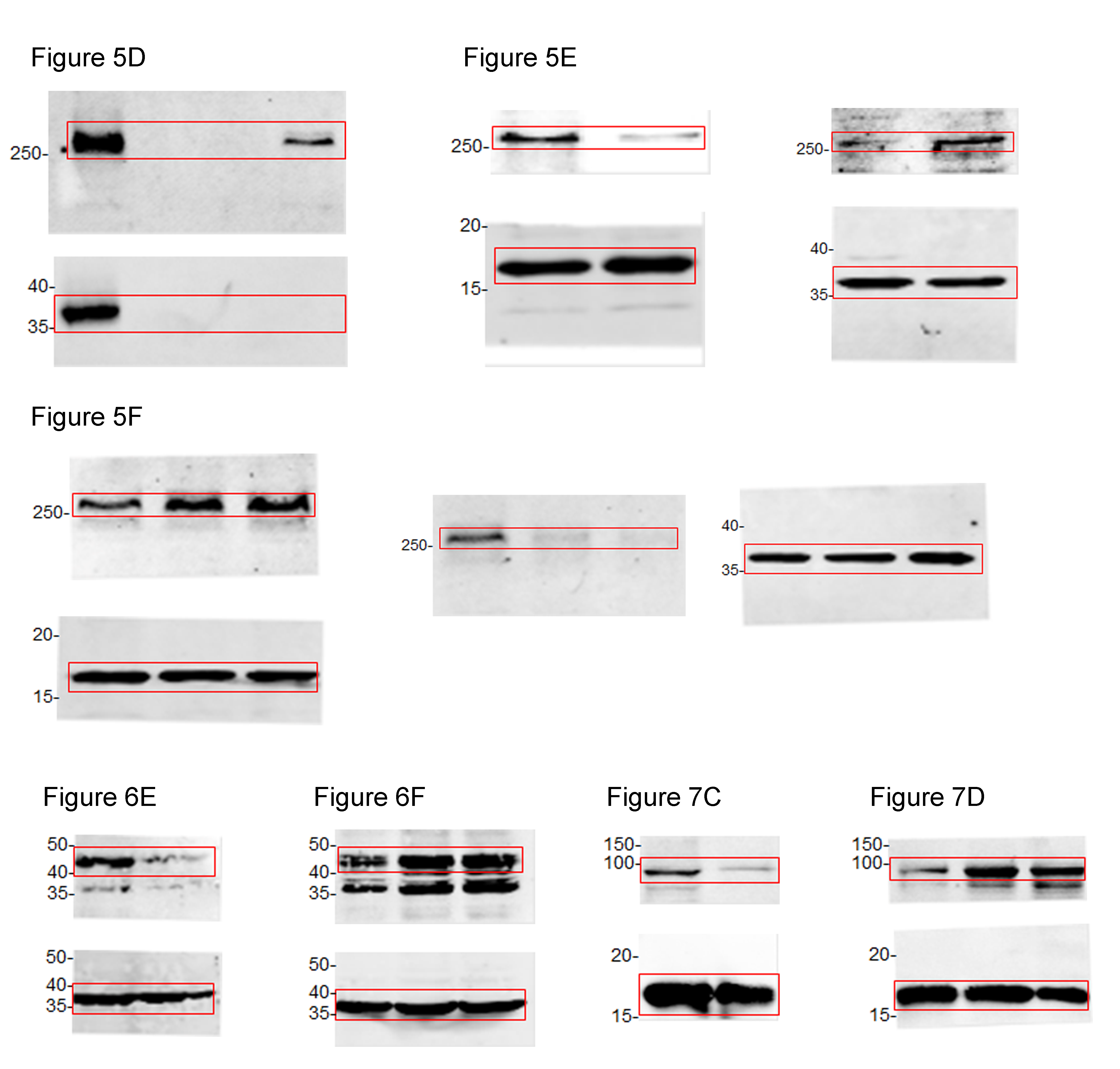

Supplement: Supplementary file 4 [file Image_4.tif]

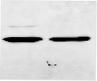

Supplement: Supplementary file 5 [file DataSheet_1.zip › western blot/Figure 5E GAPDH.tif]

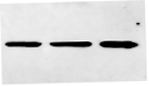

Supplement: Supplementary file 5 [file DataSheet_1.zip › western blot/Figure 5F GAPDH.tif]

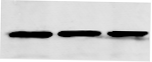

Supplement: Supplementary file 5 [file DataSheet_1.zip › western blot/Figure 5F Histone H3.tif]

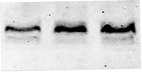

Supplement: Supplementary file 5 [file DataSheet_1.zip › western blot/Figure 5F NSD1.tif]

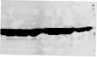

Supplement: Supplementary file 5 [file DataSheet_1.zip › western blot/Figure 6E GAPDH.tif]

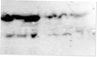

Supplement: Supplementary file 5 [file DataSheet_1.zip › western blot/Figure 6E WNT10B.tif]

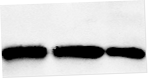

Supplement: Supplementary file 5 [file DataSheet_1.zip › western blot/Figure 7D Histone H3.tif]

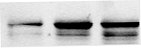

Supplement: Supplementary file 5 [file DataSheet_1.zip › western blot/Figure 7D b-catenin.tif]
